# Isovaleric Acidemia Presenting as Diabetic Ketoacidosis: A Case Report

**DOI:** 10.4274/Jcrpe.1181

**Published:** 2014-03-05

**Authors:** Mustafa Kılıç, Nazan Kaymaz, Rıza Köksal Özgül

**Affiliations:** 1 Keçiören Training and Research Hospital, Department of Pediatrics, Division of Metabolism, Ankara, Turkey; 2 Mardin Women and Children Hospital, Department of Pediatrics, Mardin, Turkey; 3 Hacettepe University Faculty of Medicine, Department of Pediatrics, Division of Metabolism, Ankara, Turkey

**Keywords:** Hyperglycemia, ketoacidosis, isovaleric acidemia

## Abstract

Isovaleric acidemia (IVA) is characterized by periodic vomiting, lethargy, coma, ketoacidosis and a ‘sweaty feet’ odor. Hyperglycemia, ketonemia, ketonuria and metabolic acidosis are the main clinical features of diabetic ketoacidosis (DKA) and these same symptoms can also be seen in acute attacks of metabolic diseases. We report a 2-year-old patient who presented with acute encephalopathy, hyperglycemia, metabolic acidosis, increased anion gap, ketosis and a preliminary diagnosis of DKA. Further investigation revealed IVA. This case is of interest because of the rarity of this presentation and detection of a splicing mutation in the isovaleryl-CoA dehydrogenase gene.

## INTRODUCTION

Isovaleric acidemia (IVA) is an autosomal recessive disease of leucine metabolism due to deficiency of isovaleryl-CoA Dehydrogenase (IVD). Acute and chronic intermittent forms have been described. Clinical manifestations in the acute form include vomiting and severe acidosis in the first few days of life, followed by progression to lethargy, convulsions, coma and death if proper therapy is not initiated. The characteristic odor of ‘sweaty feet’ may be present. In the milder chronic intermittent form, the first clinical manifestation may not appear until the child is a few months or a few years old. In both forms, an acute episode of metabolic decompensation may occur during a catabolic state such as an infection (1). IVA appearing as diabetic ketoacidosis (DKA) was reported in very few case reports (2,3,4,5,6). We considered our case to be of interest because of the rarity of presentation of this syndrome with DKA and also because of detection of a splicing mutation in the IVD gene in this patient.

## CASE REPORT

A 2-year-old female patient was admitted to our hospital with fever, vomiting, diarrhea and stupor. There was no history of psychomotor retardation nor recurrent bouts of acute encephalopathy before hospitalization. There was a first-degree consanguinity between the parents and a family history of multiple deaths with unknown etiology.

At initial assessment, the patient was lethargic and dehydrated with marked Kussmaul breathing. Blood glucose level was 334 mg/dL. Blood gas analysis revealed severe metabolic acidosis (pH:7.09) with an elevated anion gap (27.8 mmol/L) and an increased base excess (-25.2 mmol/L). Results of biochemical analyses were within normal limits except for hyperglycemia and mildly increased levels of urea, creatinine and uric acid. Ketonuria was also present (Table 1). Based on typical laboratory findings such as hyperglycemia, ketonuria, ketonemia, metabolic acidosis and clinical features such as vomiting and encephalopathy, a diagnosis of DKA was considered and treatment with intravenous fluids and insulin was started. After twelve hours of treatment, blood glucose and blood gas analysis returned to normal, but neurological status did not improve. Brain imaging studies revealed neither brain edema nor any other intracranial pathology. At that time, complete blood count examinations demonstrated pancytopenia. The patient was considered to suffer from a metabolic disease since metabolic diseases can mimic DKA. All laboratory findings were well compatible with a metabolic decompensation of an organic aciduria. Blood and urine samples were taken and saved for metabolic investigations. Erythrocyte and thrombocyte transfusions were given for the anemia and severe thrombocytopenia. Antibiotic treatment and L-carnitine (100 mg/kg IV per day) supplementations were added and a protein-restricted diet was started. Urine organic acid analysis by gas chromatography-mass spectrometry showed slight increment in concentration of 3-hydroxyisovaleric acid and a massively elevated (tenfold) concentration of isovalerylglycine. Tandem mass spectrometry of acylcarnitines in dried blood spots showed elevated C5-carnitine (isovalerylcarnitine). Mutation analysis revealed a homozygous splice site mutation (IVS4+2T>C) in the IVD gene. On the seventh day of antibiotic treatment, the patient’s fever was under control and her white blood cell and platelet counts began to increase. The clinical and neurological status of the patient was stabilized within two weeks.

## DISCUSSION

Most organic acidemias present in the newborn period as an acute episode of fulminant metabolic acidosis which may lead to coma and death if not treated adequately. Beyond the neonatal period, patients often present with developmental delay, with or without a history of recurrent acidotic episodes during catabolic stress. Typical laboratory findings during metabolic crises are metabolic acidosis, hyperammonemia, ketonuria, hypo- or hyperglycemia, anemia, thrombocytopenia, neutropenia, or pancytopenia. Untreated episodes of metabolic crises may lead to death or severe neurological sequelae (1). Metabolic decompensations can be triggered by infections, surgical intervention, dehydration, or excessive protein intake (1,7). A study by Grünert et al (8) demonstrated that gastroenteritis is the most common trigger, while protein excess or surgery did not play a major role as a precipitating factors. The first attack of metabolic crisis in our patient occurred at the age of two years and was triggered by a gastrointestinal infection. It is thought to be secondary to a viral infection. Stool examination for rotavirus antigen was negative. We could not identify the pathogen.

**Figure 1:**
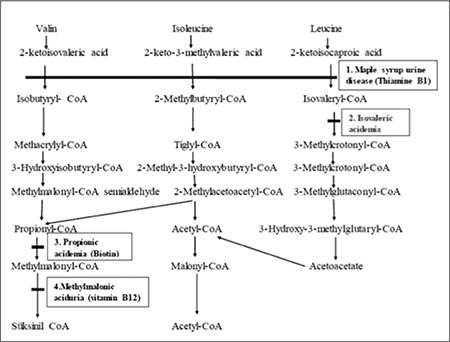
Pathways of branched-chain amino acid catabolism. 1. Branched-chain 2-keto acid dehydrogenase complex; 2. Isovaleryl-coenzyme A (CoA) dehydrogenase; 3. Propionyl-CoA carboxylase; 4. Methylmalonyl-CoA mutase. Enzyme defects are indicated by solid bars. The important metabolic diseases related to branched-chain amino acid catabolism and the enzyme cofactors used for treatment are shown in boxes ([ref:1]1[/ref][ref:][/ref])

Clinical and laboratory findings led us to an initial diagnosis of DKA. However, no improvement in encephalopathy occurred in spite of intravenous fluids and insulin therapy. These findings, in combination with the pancytopenia, the history of a first-degree cousins marriage between parents, the family history of sudden deaths of unknown cause and the sweaty feet odor, pointed towards an inborn error of metabolism. Detection of typical metabolites in blood and urine and mutation screening analyses confirmed the diagnosis of IVA (Figure 1). The pathophysiology of hyperglycemia in IVA has not yet been identified. We hypothesize that the patient may have had a transient insulin receptor defect or hypoinsulinemia due to organic acids. Insulin resistance can develop over time in patients with metabolic diseases due to acute attacks or to deficiency in insulin reserves in the pancreas. The etiology of hyperglycemia in IVA needs further investigation.

Hematologic problems can be seen in patients with inborn errors of branched-chain amino acid metabolism. Various cytopenias, attributed to the toxic effect of organic acids on hematopoietic cells and secondary heamophagocytic syndrome have been reported in IVA (9). In our patient, anemia, neutropenia and profound thrombocytopenia were observed and red blood cell and platelet transfusions were administered. After diagnosis of IVA, glucose infusion and carnitine therapy were started, in addition to antibiotics and supportive care. The diagnosis of IVA is usually based on the detection of typical metabolites of Isovaleryl-CoA in the urine organic acid analyses and of elevated C5-carnitine (isovalerylcarnitine) levels in blood and in molecular analysis (1). In our patient, direct sequencing of all coding exons and exon-intron boundaries of the IVD gene revealed a homozygous nucleotide change, namely, IVS4+2T>C. Mutation at the cryptic splice site have an effect on splicing of the IVD gene. This nucleotide change at the donor site of the intron 4 showed that it creates unstable mRNA (10).

The treatment of acute metabolic decompensation in IVA cases comprises a high-caloric infusion therapy, correction of the metabolic acidosis and supplementation with L-carnitine and L-glycine to enhance the detoxification of isovaleric acid (11).

This case illustrates that organic acidemias should be kept in mind in the differential diagnosis of DKA. Acute episodes may be misdiagnosed as DKA due to the hyperglycemia, the acidosis and presence of ketones in blood and urine.

## Figures and Tables

**Table 1:**
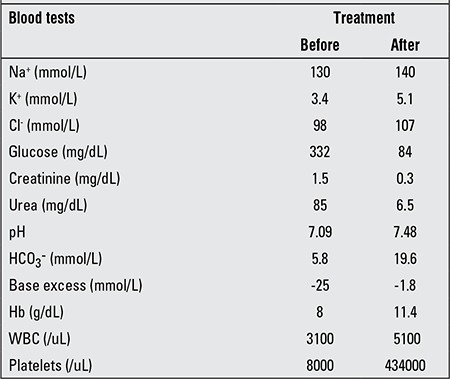
Results of blood tests before and after treatment
